# Diagnostic Value of Fine Needle Aspiration in Diagnosis of Thyroid Nodules at the Endocrine Clinic of Hamadan City During a 10-Year Period, Iran

**DOI:** 10.34172/jrhs.7768

**Published:** 2025-06-10

**Authors:** Mahsa Zamiri Mofid, Erfan Ayubi, Aidin Tarokhian, Shiva Borzouei

**Affiliations:** ^1^Department of Medicine, Faculty of Medicine, Hamadan University Medical Sciences, Hamadan, Iran.; ^2^Social Determinants of Health Research Center, Hamadan University of Medical Sciences, Hamadan, Iran; ^3^Department of Endocrinology, School of Medicine, Hamadan University of Medical Sciences, Hamadan, Iran

**Keywords:** Thyroid nodule, Fine needle aspiration, Pathology

## Abstract

**Background:** Thyroid nodules are common endocrine disorders. Most nodules are benign, with only 5% to 15% being malignant. Fine needle aspiration (FNA) is a primary diagnostic method; however, recent studies have raised concerns about its diagnostic reliability. This study aimed to evaluate performance of FNA in diagnosing thyroid nodules using pathology results as the gold standard.

**Study Design:** A cross-sectional study.

**Methods:** This study analyzed patients who were referred to an endocrine clinic in Hamadan city and underwent thyroidectomy during a 10-year period. The collected data included demographics, clinical symptoms, FNA results, and pathological outcomes. Statistical analysis was conducted using Stata software, with the significance level set at 0.05.

**Results:** The study included 700 patients, predominantly female (86.2%), with a mean age of 42.5 years. FNA results were as follows: non-diagnostic in 4.8% (n=43), benign in 43.4% (n=304), atypia of undetermined significance or follicular lesion of undetermined significance in 6.1% (n=42), follicular neoplasm in 13.2% (n=92), suspicious for malignancy in 22.8% (n=160), and malignant nodules in 9.7% (n=68) of the cases. Pathology revealed malignant nodules in 56.9% (n=398) of the cases, predominantly papillary carcinoma. Significant factors associated with malignancy included younger age, male gender, history of thyroid cancer in a first-degree relative, and the presence of cervical adenopathy. FNA showed a sensitivity of 72.43% (95% CI 67.58%, 76.93%) and a specificity of 89.64% (95% CI 85.46%, 92.95%), with an accuracy of 79.85% (95% CI 76.55%, 82.87%).

**Conclusion:** While FNA is a valuable diagnostic tool for thyroid nodules, its sensitivity varies, necessitating close follow-up of patients with negative results.

## Background

 Thyroid nodules are prevalent endocrine disorders characterized by localized growth and proliferation of thyroid cells within the thyroid gland. Most nodules are asymptomatic and are typically detected during head and neck imaging.^1,2^ Epidemiological studies indicate that the prevalence of these nodules in adults ranges from 5% to 7% through physical examination and up to 19% to 68% through imaging techniques such as neck ultrasonography. The incidence of thyroid nodules is greater in women and elderly individuals.^3-5^ Factors contributing to increased incidence of nodules include smoking (particularly in areas with mild iodine deficiency), obesity, metabolic syndrome, alcohol consumption, elevated insulin-like growth factor levels, and the presence of uterine fibroids.^6-10^

 Thyroid nodules can be categorized as benign or malignant. Approximately 90% to 95% of thyroid nodules are benign, with only 5% to 15% being malignant, predominantly papillary thyroid carcinoma.^11,12^ According to the World Health Organization statistics, thyroid cancer was diagnosed in 586 000 patients worldwide in 2020, ranking ninth in incidence.^13^

 Risk factors that increase the risk of malignancy in thyroid nodules include younger age, a history of exposure to ionizing radiation, and a family history of thyroid cancer in first-degree relatives.^14^ Clinical signs suggestive of malignancy include cervical adenopathy, vocal cord paralysis, and hoarseness. Ultrasound features indicative of malignancy include solid nodules, hypoechogenicity, a nodule size larger than 4 cm, a greater length-to-width ratio, microcalcifications, internal vascularity, and irregular margins.^15,16^

 Fine needle aspiration (FNA) is the preferred diagnostic method for thyroid malignancies due to its high accuracy, safety, simplicity, and cost-effectiveness. Cytological findings from FNA are used to classify thyroid nodules, guiding decisions for surgery or follow-up. Despite advancements in diagnostic methods, FNA remains the primary tool for distinguishing between benign and malignant nodules.^17,18^

 FNA results are categorized according to the Bethesda system, with the likelihood of malignancy in the groups ranging from 5% to 10% (I), 0% to 3% (II), 10% to 30% (III), 25% to 40% (IV), 50% to 75% (V), and 97% to 99% (VI). The Bethesda system aims to provide a standardized approach for estimating malignancy risk and selecting appropriate candidates for surgery.^19^

 Studies have reported that the accuracy, sensitivity, specificity, and false-negative rate of thyroid FNA, particularly under ultrasound guidance, are 95%, 89%-98%, 92%, and less than 5%, respectively.^20-23^ The diagnostic performance of FNA is influenced by nodule size and specific ultrasound features. When performed by an experienced physician, FNA yields reliable information; however, cytological studies may be inconclusive due to insufficient material or a lack of morphological diagnostic criteria.^24^

 Recent studies have highlighted an increase in both the false-negative rate and false-positive rate, especially for large nodules, raising concerns about the diagnostic reliability of FNA. False-negative FNA results can delay diagnosis and treatment.^25-27^ Consequently, further research is essential to evaluate the diagnostic value of FNA and associated factors in determining the malignancy of thyroid nodules after thyroidectomy and pathological results.

 This study aimed to evaluate the performance of FNA in thyroid nodules using pathology as the gold standard. The findings can highlight the limitations of this widely used method and offer valuable insights to support more comprehensive research in the future.

## Materials and Methods

 This cross-sectional study was conducted at an endocrine clinic in Hamadan city from 2013 to 2023. The study population included all individuals who visited the clinic for evaluation of thyroid nodules and underwent thyroidectomy over a 10-year period. Only patients with differentiated thyroid cancer were included in the study. Patients with insufficient medical records or follow-up were excluded from the study.

 Following the approval of the research proposal and obtaining permission from the Ethics Committee of Hamadan University of Medical Sciences, a checklist was utilized to collect demographic information (age and gender), medical history (family history of thyroid diseases (benign or malignant), history of exposure to radiation, type of thyroid disease including euthyroid, clinical hypothyroidism, clinical hyperthyroidism, subclinical hypothyroidism, and subclinical hyperthyroidism), presence of clinical symptoms (mass symptoms, compressive symptoms, obstructive symptoms, voice hoarseness, and vocal cord paralysis), and physical examination details (solitary or multiple nodules, size of nodules, and presence of cervical adenopathy) prior to surgery. FNA results, categorized according to the Bethesda system, and pathology results (benign and malignant) were also recorded. All pathology slides were evaluated by two pathologists. The pathologists remained the same for each patient.

 The Bethesda System for Reporting Thyroid Cytopathology is a classification system for thyroid FNA biopsy results. It includes six diagnostic categories: (I) non-diagnostic or unsatisfactory (insufficient sample for diagnosis, with a malignancy risk of 1%-4%), (II) benign (non-cancerous findings, with a malignancy risk of 0%-3%), (III) atypia of undetermined significance or follicular lesion of undetermined significance (indeterminate findings, with a malignancy risk of 5%-15%), (IV) follicular neoplasm or suspicious for follicular neoplasm (possible neoplasm, with a malignancy risk of 15%-30%), (V) suspicious for malignancy (strong suspicion of cancer, with a malignancy risk of 60%-75%), and (VI) malignancy (diagnostic of cancer, with malignancy risk of 97%-99%) (19).

 This system provides a standardized approach for interpreting thyroid cytology and guiding clinical management.

 Descriptive statistics, including the mean ( ± standard deviation) and frequency (percentage), were used to describe quantitative variables. The chi-square test was employed to assess the relationships between qualitative variables. Independent *t* test was used to compare quantitative variables. Diagnostic indices evaluated were sensitivity, specificity, accuracy, positive predictive value, negative predictive value, positive likelihood ratio, negative likelihood ratio, area under receiver-operator curve, and f1 score (harmonic mean of positive predictive value and sensitivity). Statistical analysis was conducted using Stata version 14 software, with the significance level set at 0.05. The results were presented with 95% confidence intervals.

## Results

 A total of 700 surgically operated patients who met the study criteria were analyzed. The mean age of the patients was 42.5 ± 13.5 years. Among these patients, 97 (13.8%) were male, and 603 (86.2%) were female. History of thyroid disease in first-degree relatives was reported by 27.4% (n = 192) of the patients, 14.6% (n = 28) of whom had a history of thyroid cancer in first-degree relatives. Additionally, 42.8% (n = 300) of the patients exhibited clinical signs.

 In most patients (82.6%, n = 578), thyroid function was normal. The most prevalent thyroid disorder was subclinical hypothyroidism (5.1%, n = 36), followed by clinical hyperthyroidism (4.6%, n = 32). During thyroid examination, a solitary thyroid nodule was observed in 74.3% (n = 520) of the patients. Nodules smaller than 4 cm were present in 72.6% (n = 508) of the patients. Cervical adenopathy was absent in 81.7% (n = 572) of the patients and it was present in 18.3% (n = 128).

 The FNA results were as follows: non-diagnostic in 4.8% (n = 34), benign in 43.4% (n = 304), atypia of undetermined significance or follicular lesion of undetermined significance in 6.1% (n = 42), follicular neoplasm in 13.2% (n = 92), suspicious for malignancy in 22.8% (n = 160), and malignancy in 9.7% (n = 68) of the cases. Pathological results revealed benign results in 43.1% (n = 302) of patients and malignant results in 56.9% (n = 398) (Table 1).

**Table 1:** Demographic information of patients

**Category**	**Frequency**	**Percent**
Gender		
Male	97	13.8
Female	603	86.2
Thyroid disease in the first-degree relatives		
Yes	192	27.4
Benign	164	85.4
Malignant	28	14.6
No	508	72.6
Symptoms		
Present	300	42.8
Absent	400	57.2
Thyroid function		
Euthyroid	578	82.6
Hypothyroidism	29	4.2
Subclinical hypothyroidism	36	5.1
Hyperthyroidism	32	4.6
Subclinical hyperthyroidism	25	3.5
Solitary or multiple		
Solitary nodule	520	74.3
Multiple nodules	180	25.7
Nodule size		
Smaller than 4 cm	508	72.6
4 cm or larger	192	27.4
Cervical adenopathy		
None	572	81.7
Present	128	18.3
Fine-needle aspiration results		
Non-diagnostic or unsatisfactory	34	4.8
Benign	304	43.4
AUS/FUS	42	6.1
FN/SFN	92	13.2
Suspicious for malignancy	160	22.8
Malignancy	68	9.7
Pathology results		
Benign	302	43.1
Malignant	398	56.9

AUS/FUS: Atypia of undetermined significance or follicular lesion of undetermined significance; FN/SFN: Follicular neoplasm or suspicious for follicular neoplasm.

**Figure 1:**
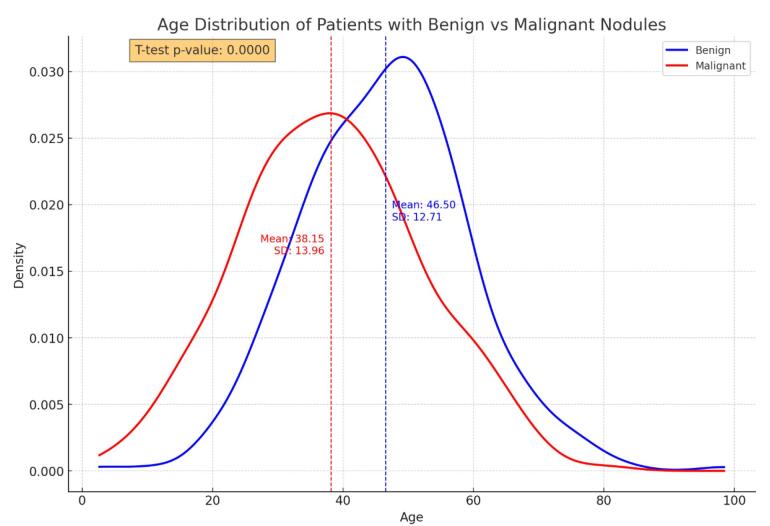
A comparison of the age distribution between patients with benign and malignant thyroid nodules based on pathological results

 The average age of patients with malignant nodules was significantly lower than that of patients with benign masses (difference of 3.9 years, *P* < 0.001) (Figure 1).

 Additionally, 70% of the thyroid nodules in men were malignant, compared to 54.8% in women (*P* = 0.007). The frequency of a family history of thyroid cancer in a first-degree relative was significantly greater in the malignant group than in the benign group (*P* < 0.001). The presence of clinical symptoms did not significantly differ between benign and malignant patients (*P* = 0.173). The frequency of functional thyroid disorders was significantly greater in benign cases than in malignant cases (*P* < 0.001).

 In malignant cases, solitary nodules were predominant, whereas in benign cases, multiple nodules were more prevalent, and this difference was statistically significant (*P* < 0.001). Most nodules in both groups were smaller than 4 cm, with no statistically significant difference (*P* = 0.904).

 The frequency of adenopathy was greater in patients with malignancies than in those with benign conditions (*P* < 0.001). There was a statistically significant difference between the pathology results and the FNA findings (*P* < 0.001). A detailed comparison between them is presented in Table 2.

**Table 2:** Comparison of categorical variables between patients with malignant and benign nodules

**Variables**	**Benign nodule, n=302**		**Malignant nodule, n=398**		***P *** **value**
	**Number**	**Percent**	**Number**	**Percent**	
Gender					0.007
Male	29	9.6	68	17	
Female	273	90.4	330	83	
History of malignancy in the first-degree relatives					0.001
Present	6	2.1	22	5.5	
Absent	296	97.9	376	94.5	
Symptoms					0.173
Present	136	45	164	41.1	
Absent	166	55	234	58.9	
Thyroid function					0.001
Abnormal	79	26.1	43	10.8	
Normal	223	73.9	355	89.2	
Nodule					0.001
Solitary	193	63.9	327	82.2	
Multiple	109	36.1	71	17.8	
Nodule size (cm)					0.904
≤ 4	220	72.9	288	72.4	
> 4	82	27.1	110	27.6	
Adenopathy					0.001
Present	14	4.6	114	28.6	
Absent	288	95.4	284	71.4	

 The sensitivity of FNA was 72.43% (95% CI: 67.58% to 76.93%). The specificity was 89.64% (95% CI: 85.46% to 92.95%). The positive likelihood ratio was 6.99 (95% CI: 4.93 to 9.93), and the negative likelihood ratio was 0.31 (95% CI: 0.26 to 0.36). The positive predictive value was 90.24% (95% CI: 86.69% to 92.92%), and the negative predictive value was 71.10% (95% CI: 67.49% to 74.47%). The accuracy of the FNAs was 79.85% (95% CI: 76.55% to 82.87%). The performance metrics of FNA are presented in Table 3.

**Table 3:** Different performance metrics of FNA

**Metric**	**Value % (95% CI)**
Sensitivity	72.43 (67.58, 76.93)
Specificity	89.64 (85.46, 92.95)
Positive likelihood ratio	6.99 (4.93, 9.93)
Negative likelihood ratio	0.31 (0.26, 0.36)
Positive predictive value	90.24 (86.69, 92.92)
Negative predictive value	71.10 (67.49, 74.47)
Accuracy	79.85 (76.55, 82.87)
F1 score	0.80 (not applicable)
ROC-AUC	0.81 (not applicable)

ROC-AUC: Receiver operating curve-area under the curve. F1 score is a harmonic mean of positive predictive value and sensitivity.

## Discussion

 In the present study, the FNA method exhibited limitations in diagnosing malignant and benign cases. However, its specificity and positive predictive value are adequate for diagnosis. In other studies, the sensitivity of FNA has been reported to range from 35% to 93%, which may be attributed to differences in the size of thyroid nodules. Previous studies have shown that the sensitivity of the FNA method may be influenced by the size of the thyroid nodules. Studies including patients with a nodule size equal to or greater than 4 cm have reported higher sensitivity for this method.^24,28-31^ In this study, we did not analyze the FNA results concerning nodule size. This omission could partially explain the discrepancies between different studies.

 Another reason for the variability in the sensitivity of FNA may be the limited accuracy of the method for distinguishing benign lesions such as follicular adenomas and hyperplastic follicular nodules from malignant follicular carcinoma.^32^

 In one study, pathological findings after thyroidectomy revealed thyroid cancer in 100% of the patients classified as malignant on FNA, 33.3% of the patients classified as suspicious for malignancy, 7.7% of the patients suspected of follicular neoplasm, 17.6% of the patients classified as atypical or follicular, and 4.1% of the patients classified as benign on FNA. No thyroid cancer was diagnosed in any of the non-diagnostic patients.^33^ Both studies showed consistency in terms of the pathological results and the FNA findings; however, there was a slight discrepancy between the two studies in the other categories, which may be due to differences in the study population, the FNA procedure, and the expertise of the physicians performing the FNA.

 Based on the results, FNA can be considered a simple, cost-effective, and non-invasive diagnostic method for thyroid lesions that can significantly impact patient management and cost-effectiveness. However, considering the limitations of the FNA method in diagnosing benign lesions, the possibility of false-negative results, and the slow growth of thyroid cancer, patients with non-malignant findings based on FNA should undergo periodic follow-up.

 This study also highlighted several historical and physical examination findings that are useful for differentiating between benign and malignant thyroid nodules. While older age is associated with an increased incidence of thyroid nodules, younger age is linked to a greater likelihood of malignancy. Additional factors associated with malignancy include male gender, the presence of a solitary nodule, and cervical adenopathy. This finding is compatible with the findings of other studies.^34,35^

 The main limitations of the study include its retrospective design and its restriction to the specialized endocrine clinic in Hamedan city. As a result, the generalizability of the findings of the study to patients in other areas is limited (possible selection bias).

**Highlights**

- Based on pathology results, 56.9% of thyroid nodules were malignant.
- The sensitivity of FNA was 72.43% and its specificity was 89.64%.
- FNA is a valuable diagnostic tool for thyroid nodules.

## Conclusion

 The findings revealed that while FNA has high specificity (89.64%) and positive predictive value (90.24%), its sensitivity (72.43%) and negative predictive value (71.10%) are limited. Given the potential for false-negative results, it is crucial for patients with non-malignant FNA findings to undergo regular follow-up to monitor for any changes. Despite its limitations, FNA remains a cost-effective and minimally invasive diagnostic tool. However, further research is necessary to enhance its reliability and reduce diagnostic discrepancies.

## Acknowledgments

 The authors would like to thank the Research Ethics Committee of Hamadan University of Medical Sciences. The work was submitted under the code 140103242117.

## Competing Interests

 The authors have no conflict of interests.

## Ethical Approval

 This study was conducted in accordance with the principles of the Helsinki Declaration and was approved by the Ethics Committee of Hamadan University of Medical Sciences (IR.UMSHA.REC.1401.099).

## Funding

 No financial support was provided for this study.
